# Development of multidisciplinary, evidenced-based protocol recommendations and implementation strategies for anterior lumbar interbody fusion surgery following a literature review

**DOI:** 10.1097/MD.0000000000036142

**Published:** 2023-11-24

**Authors:** Richard Meyrat, Elaina Vivian, Archana Sridhar, R. Heath Gulden, Sue Bruce, Amber Martinez, Lisa Montgomery, Donald N. Reed, Peter J. Rappa, Hetendra Makanbhai, Kenneth Raney, Jennifer Belisle, Stacey Castellanos, Judy Cwikla, Kristin Elzey, Kristen Wilck, Fallon Nicolosi, Michael E. Sabat, Chris Shoup, Randall B. Graham, Stephen Katzen, Bartley Mitchell, Michael C. Oh, Nimesh Patel

**Affiliations:** a Methodist Moody Brain and Spine Institute, Methodist Health System, Dallas, TX; b Performance Improvement, Methodist Dallas Medical Center, Dallas, TX; c Anesthesia Consultants of Dallas Division, US Anesthesia Partners, Dallas, TX; d Clinical Outcomes Management, Methodist Dallas Medical Center, Dallas, TX; e Pre-Surgery Assessment, Methodist Dallas Medical Center, Dallas, TX; f Neurosurgery Division, Methodist Health System, Dallas, TX; g Rehab Medicine Associates, Dallas, TX; h Dallas Hospitalists PA, Dallas, TX; i Pharmacy, Methodist Dallas Medical Center, Dallas, TX; j Neurocritical Care Unit, Methodist Dallas Medical Center, Dallas, TX; k Clinical Nutrition, Methodist Dallas Medical Center, Dallas, TX; l Methodist Community Pharmacy – Dallas, Methodist Dallas Medical Center, Dallas, TX; m Surgery and Recovery, Methodist Dallas Medical Center, Dallas, TX; n Executive Office, Methodist Health System, Dallas, TX.

**Keywords:** anterior lumbar interbody fusion, evidence-based recommendation, perioperative care, postoperative ileus, postoperative pain management

## Abstract

The anterior lumbar interbody fusion (ALIF) procedure involves several surgical specialties, including general, vascular, and spinal surgery due to its unique approach and anatomy involved. It also carries its own set of complications that differentiate it from posterior lumbar fusion surgeries. The demonstrated benefits of treatment guidelines, such as Enhanced Recovery after Surgery in other surgical procedures, and the lack of current recommendations regarding the anterior approach, underscores the need to develop protocols that specifically address the complexities of ALIF. We aimed to create an evidence-based protocol for pre-, intra-, and postoperative care of ALIF patients and implementation strategies for our health system. A 12-member multidisciplinary workgroup convened to develop an evidence-based treatment protocol for ALIF using a Delphi consensus methodology and the Grading of Recommendations, Assessment, Development, and Evaluation (GRADE) system for rating the quality of evidence and strength of protocol recommendations. The quality of evidence, strength of the recommendation and specific implementation strategies for Methodist Health System for each recommendation were described. The literature search resulted in 295 articles that were included in the development of protocol recommendations. No disagreements remained once the authors reviewed the final GRADE assessment of the quality of evidence and strength of the recommendations. Ultimately, there were 39 protocol recommendations, with 16 appropriate preoperative protocol recommendations (out of 17 proposed), 9 appropriate intraoperative recommendations, and 14 appropriate postoperative recommendations. This novel set of evidence-based recommendations is designed to optimize the patient’s ALIF experience from the preoperative to the postoperative period.

## 1. Introduction

Anterior lumbar interbody fusion (ALIF) is a form of spinal fusion that utilizes the anterior retroperitoneal approach to expose the entire ventral surface of the intervertebral space. ALIF results in better correction of coronal imbalance, greater restoration of lordosis, and higher fusion rates than other approaches.^[1–5]^ ALIF also allows for sparing of the posterior paraspinal ligaments and muscles, maintains the posterior tension band for postural support, and reduces postoperative pain from muscle dissection.^[6]^ Over the last couple of decades, technical advances and improved surgical training have greatly improved the safety and efficacy of the ALIF procedure, which can include standalone ALIF, where no supplemental posterior fusion or fixation is utilized, and ALIF with transpedicular instrumented fusion (i.e., ALIF 360). For many surgeons, it has become the preferred approach for L4-5 and L5-S1 interbody fusions.

However, due to its unique approach and anatomy involved, the ALIF procedure crosses over several surgical specialties, including general, vascular, and spinal surgery. It also carries its own set of complications that differentiate it significantly from posterior lumbar fusion surgeries. Most of the complications of ALIF are related to the approach, such as vessel injury, ileus, hernia, ureteral injury, and retrograde ejaculation.^[7–12]^ Risk of vascular injury is the main reason many spinal surgeons work with vascular or spinal access surgeons during the exposure. Occurrence rates of vascular injury vary considerably, from 1.9% to 24%^[13–15]^ or as high as 55% in some series.^[14]^ The most common type of vascular injury is venous laceration,^[16]^ but could also include aortic thrombosis^[16]^ or aortic dissection.^[15]^ Deep vein thrombosis (DVT) is another potential complication, with incidence rates ranging from 0% to 5%.^[15]^ The management of postoperative ileus (POI) falls under general surgery management, and the rate of POI after ALIF has been reported to be between 3% and 5.44%.^[17,18]^ Gerbershagen et al^[19]^ conducted a review that found spinal surgeries were one of the top 10 procedures with the highest degree of postsurgical pain. Poorly managed postsurgical pain can then lead to other complications like venous thrombosis, respiratory complications, or development of chronic functional deficits.^[20]^ Overall, ALIF complications like vascular injury, POI, and inadequately treated postsurgical pain can lead to prolonged length of stay, higher costs, and decreased satisfaction in patients.

Advances in surgical techniques and instrumentation will continue to propel ALIF’s popularity. There has been an average annual increase of 24.07% for ALIF procedures, with the most common associated pathology being degenerative disc disease and spondylolisthesis where a third of patients undergo multilevel fusion.^[21]^ This fact combined with ALIF’S unique challenges and the incorporation of various surgical specialties highlights the need for treatment pathways that support improved outcomes for patients. Enhanced Recovery after Surgery (ERAS) pathways have been shown across a variety of surgical specialties to enable “shorter length of stay, decreased postoperative pain and need for analgesia, decreased complication and readmission rates, and increased patient satisfaction.^[22]^” In regards to spine surgery, the ERAS Society has published guidelines for the perioperative care in lumbar spinal fusion,^[23]^ but these guidelines effectively excluded ALIF.

The demonstrated benefits of ERAS in other surgical procedures and the lack of current recommendations regarding the anterior approach underscores the need to develop protocols that specifically address the complexities of standalone and ALIF 360s.^[23]^ As a result, Methodist Health System and the Moody Brain and Spine Institute assembled a multidisciplinary workgroup in order to develop an evidence-based treatment protocol for ALIF using a Delphi consensus methodology^[24]^ and the Grading of Recommendations, Assessment, Development, and Evaluation (GRADE) system for rating the quality of evidence and strength of protocol recommendations.^[25]^

The key research question/objective was to evaluate existing evidence, which is applicable to the perioperative management of ALIF patients, to support protocol recommendations intended to improve ALIF outcomes. The protocol recommendations cover 3 domains corresponding to the different phases of surgery: preoperative, intraoperative, and postoperative. Although broad, evidence-based recommendations were established to optimize the patient’s surgical experience from the preoperative to the postoperative period, we also developed specific strategies to implement the ALIF protocol at our hospital. Both general recommendations and our corresponding treatment strategies are presented in this paper in the results section.

## 2. Methods

The ALIF-specific protocol was developed using the RAND/UCLA Appropriateness Methodology, a well-defined and scientifically rigorous consensus methodology. This methodology enables the combination of evidence-based recommendations with the collective judgment of clinical experts in their respective specialties and to determine appropriateness and validity of diagnostic and treatment options in clinical care.^[24]^ The study exempt from Institutional Review Board review, as it didn’t qualify as human subjects research.

### 2.1. Establishment of the multidisciplinary workgroup/expert panel

The RAND/UCLA Appropriateness Methodology recommends the use of multidisciplinary teams to reflect the variety of specialties involved in treatment decisions for patients. Our workgroup/expert panel included neurosurgeons, access/general surgeons, anesthesiologists, internists, a pain management specialist, a physiatrist, neurosurgery nurse practitioners, pharmacists, nutritionists, a nurse manager of the neuro critical care unit, and a registered nurse-ERAS program coordinator. The workgroup was advised that this initiative would focus on ALIF with an emphasis on protocol recommendations to reduce the risk of ileus and postoperative pain. Suggestions for recommendations and specific implementation strategies were taken from the entire workgroup and then evaluated and decided upon by all members. The final paper was agreed upon by all authors.

### 2.2. Literature search, evidence summaries, and GRADE classification

A systematic literature review was conducted in accordance with the Preferred Reporting Items for Systematic Review and Meta-Analysis Protocols Statement.^[26]^ The review consisted of searching PubMed, Google Scholar, Cochrane Library, and ERAS Society Guidelines website to identify consensus guidelines, randomized control trials, meta-analyses, observational studies, and expert opinion pieces related to lumbar fusions, ALIF, other abdominal approach surgeries with similar considerations to ALIF (e.g., colorectal, general, vascular, etc.), and pieces related to ALIF-related complications (e.g., ileus, incisional hernias, etc.). The search strategies were created using medical subject headings and non-medical subject headings terms and keywords, and there were no limits on the date of publication. Reference lists of some articles were also checked for other relevant studies. This produced 479 records to be screened. Non-English studies, duplicates, full-text reports that could not be retrieved, and irrelevant reports were excluded. Ultimately, 295 studies were included in our review. Although the literature review conducted was not exhaustive, it was intended to represent an overview of evidence on key concepts/recommendations, identify areas that deserve further clarification, and discover opportunities for future research. The proposed protocol recommendations were organized across 3 main domains commonly found in ERAS guidelines: pre-, intra-, and postoperative.

Evidence summaries for each proposed recommendation were developed and included the domain, clinical aspects/implementation details, rationale, exclusions/contraindications, a list of the supporting literature, and each article’s methodological approach. The evidence summaries were then used to apply the GRADE framework to evaluate supporting literature and identify the quality of evidence (Table 1) and strength of each recommendation (Table 2).^[25]^ The application of GRADE was initially completed by the neurosurgeons and research staff and then shared with the entire workgroup for consensus review and approval.

**Table 1:** Grading of Recommendations, Assessment, Development, and Evaluation system for rating quality of evidence.^[25]^

Evidence level	Definition
High quality	Further research unlikely to change confidence in estimate of effect
Moderate quality	Further research likely to have important impact on confidence in estimate of effect and may change the estimate
Low quality	Further research very likely to have important impact on confidence in estimate of effect and likely to change the estimate
Very low quality	Any estimate of effect is very uncertain

**Table 2:** Grading of Recommendations, Assessment, Development, and Evaluation system for rating strength of recommendation.^[25]^

Recommendation strength	Definition
Strong	When desirable effects of intervention clearly outweigh the undesirable effects, or clearly do not
Weak	When trade-offs are less certain-either because of low quality evidence of because evidence suggests desirable and undesirable effects are closely balanced

### 2.3. Consensus process

Upon reviewing the literature compiled, proposed protocol recommendations and evidence summaries were sent to the workgroup via email. Recipients were then asked to carefully review the information and rate the proposed protocol recommendations in 2 rounds (for each of the 3 domains).

In round one, workgroup members performed an independent review and rated the recommendations for appropriateness on a 9-point Likert scale, where 1 = definitely not appropriate and 9 = definitely appropriate. In round two, a panel further vetted each recommendation in person and reviewed the aggregated data for appropriateness from round one. Modifications were made according to consensus. Once each recommendation was discussed, the panel rated the recommendations again for appropriateness on the 9-point scale.

### 2.4. Statistical analysis and classification of recommendations

Appropriateness was classified as appropriate or not appropriate based on the median rating and whether workgroup members agreed, as measured by the amount of dispersion of the ratings. Appropriate recommendations required a median rating between 7 and 9. To determine statistical agreement (or the dispersion of ratings), the BIOMED classical definition of agreement was used, which sets the maximum number of responses that are allowed to fall outside the 3-point region (i.e., 1–3, 4–6, and 7–9) containing the median to conclude agreement.^[24]^

### 2.5. Implementation strategies

After the final set of protocol recommendations were developed, the workgroup re-convened to discuss specific implementation strategies for Methodist Health System. These included multimodal interventions (e.g., specific order sets detailing the timing and routes of administration). This process relied heavily on the expert opinion of the workgroup and was supported by evidence, when available.

## 3. Discussions/observations

A 12-member multidisciplinary workgroup was convened to develop protocol recommendations. Ultimately, there were 39 total protocol recommendations, with 16 preoperative protocol recommendations (out of 17 proposed), 9 intraoperative recommendations, and 14 postoperative recommendations (see Table S1, Supplemental Digital Content, http://links.lww.com/MD/K785, which illustrates the 39 protocol recommendations).

The literature search resulted in 295 articles that were included in the development of protocol recommendations. No disagreements remained once the authors reviewed the final GRADE assessment of the quality of evidence and strength of the recommendations.

### 3.1. Protocol recommendations

#### 3.1.1. Patient education and counseling.

Preoperative patient education is an important component of current ERAS guidelines.^[23,27,28]^ Managing patients’ expectations can reduce fear and anxiety related to uncertainty in surgical outcomes and postoperative complications.^[29]^ Patients whose expectations were met through adequate education about postoperative pain were found to be more satisfied with their care.^[30]^ This is important because lumbar surgery is often painful which can lead to a longer recovery time. Although several randomized controlled trials (RCTs) have demonstrated limited evidence for the effectiveness of preoperative education and counseling on reducing postoperative complications,^[31]^ we recommend preoperative education for patients undergoing ALIF given its low risk profile and promising benefits. Further studies are warranted in this area to determine the timing and methods of delivery of patient education.

Discharge education can lead to reduced postoperative emergency department visits and hospital readmissions^[32]^ by helping patients manage their postoperative course once discharged home. Patient-oriented education in the form of pamphlets or booklets can teach patients how to recognize postoperative complications and how to perform physical activities that contribute to a safe and effective rehabilitation. In addition, it can provide information on medical management, diet, and home environment management.^[33]^ Providing patients with the appropriate contact information in the event of a concern or complication can help reduce patient anxiety and prevent unnecessary emergency department visits.^[32]^ As in the case of preoperative education, there is limited evidence that discharge education is effective despite its ostensible benefits and low risks. Further research in this area is required to establish its effectiveness (Table 3).

**Table 3:** Patient education and counseling recommendations and implementation strategies.

	GRADE
Preoperative recommendation	
Preoperative patient education is recommended	Quality of evidence: Moderate Recommendation Grade: Strong
Preoperative implementation strategy	
Provide patients a written or electronic education pamphlet regarding ALIF surgery	
Postoperative recommendation	
Discharge education is recommended	Quality of evidence: Low Recommendation Grade: Strong
Postoperative implementation strategy	
In addition to the education pamphlet, the patient will be given discharge instructions regarding postoperative care and will be provided time for questions and concerns prior to discharge	

ALIF = anterior lumbar interbody fusion.

#### 3.1.2. Prehabilitation.

Prehabilitation refers to enhancing the functional capacity of a patient in preparation for an anticipated surgical procedure.^[34]^ Targeted physical and cognitive interventions are given by combining exercise, nutritional therapy and psychological preparation. Prehabilitation is recommended for a period of 4 weeks before surgery.^[35]^ Prehabilitation in patients undergoing abdominal, cardiac, or vascular surgery results in fewer postoperative complications with reduced length of stay and improved health-related quality of life compared to control groups.^[36–38]^ There is insufficient evidence that prehabilitation in patients undergoing orthopedic and spine surgery improves functional outcomes after surgery^[39]^; however, we recommend it for all patients undergoing ALIF. Further research is required to determine the efficacy of prehabilitation and to ascertain specific patient groups that would benefit the most from it (Table 4).

**Table 4:** Prehabilitation recommendations and implementation strategies.

	GRADE
Preoperative recommendation	
Prehabilitation combining exercise, nutritional therapy, and psychological preparation is recommended	Quality of evidence: Very low Recommendation Grade: Weak
Preoperative implementation strategy	
As above for a minimum of 4 wk	

#### 3.1.3. Preoperative smoking cessation.

Tobacco smoking is associated with unfavorable surgical outcomes, causing increased incidence of wound infections, sepsis, pseudoarthrosis, morbidity, and mortality.^[40]^ It is also considered to be an independent risk factor for postoperative ileus.^[39]^ Preoperative smoking cessation interventions are recommended for a minimum of 4 weeks before surgery and to be continued after surgery.^[41–43]^ A meta-analysis of RCTs among patients undergoing various elective surgeries showed that each week of smoking cessation increased the magnitude of treatment effect by 19%.^[41]^ Interventions include nicotine replacement therapy combined with intensive counseling for both short- and long-term benefits. Patients undergoing spine surgery should particularly be counseled on smoking because it is associated with increased recurrence of lumbar disc herniation, postoperative opioid requirement, and pseudoarthrosis (Table 5).^[44–46]^

**Table 5:** Preoperative smoking cessation recommendations and implementation strategies.

	GRADE
Preoperative recommendation	
Smoking cessation interventions are recommended at a minimum of 4 wk before surgery	Quality of evidence: Moderate Recommendation Grade: Strong
Preoperative implementation strategy	
All patients will be asked about their smoking status preoperatively. A minimum period of 4 wk of cessation is recommended. Nicotine replacement therapy combined with intensive counseling will be provided. Smokers will be counseled about the increased risks of surgery	

#### 3.1.4. Preoperative alcohol cessation.

High-risk drinking is defined as alcohol consumption equivalent to more than 3 alcoholic units (AU)/d or 21 AU/wk (with 1 AU containing 12 g of ethanol) with or without symptoms of alcohol abuse or dependency.^[47]^ It is associated with increased postoperative complications such as infections, cardiopulmonary complications, and bleeding.^[47]^ Meta-analyses and systemic reviews have shown excess drinking leads to increased adverse events after spinal surgery.^[48,49]^ Alcohol cessation interventions involve a combination of pharmacological and psychosocial strategies, such as disulfiram, benzodiazepines, vitamins, and behavioral modifications.^[47]^ Meta-analyses of RCTs involving patients undergoing orthopedic and neurosurgical procedures have shown that alcohol cessation intervention for a minimum of 4 to 8 weeks before surgery was most effective in reducing complications.^[47,50,51]^ These studies demonstrated moderate quality evidence despite being limited by small sample size and a lack of long-term follow-up (Table 6).^[47]^

**Table 6:** Preoperative alcohol cessation recommendations and implementation strategies.

	GRADE
Preoperative recommendation	
Alcohol cessation interventions are recommended at a minimum of 4–8 wk before surgery to reduce postoperative complications	Quality of evidence: Moderate Recommendation Grade: Strong
Preoperative implementation strategy	
All patients will be asked about their alcohol consumption habits. Patients identified as high-risk drinkers will be referred for preoperative alcohol cessation 4–8 wk before surgery. Alcohol cessation interventions may include a combination of behavioral modifications, disulfiram, vitamins, and benzodiazepines. A preoperative liver function panel will be ordered for all patients	

#### 3.1.5. Preoperative diabetes monitoring.

Diabetes is a chronic disease with a prevalence of 10.5% (34.2 million) in the United States in 2018.^[52]^ In 1 case-control study, the incidence of diabetes in patients with lumbar spine stenosis was 29.1%.^[53]^ Uncontrolled diabetes is correlated with higher rates of complications after surgery including increased risk of infections, length of stay, higher healthcare costs, and mortality.^[54]^ Preoperative investigations should include HbA1c (drawn within the last 3 months) if the patient has diabetes or has a blood glucose level > 120 mg/dL. There is no published evidence-based guideline that precludes surgery above a particular HbA1c level. However, levels below 8% to 9% is considered a safe target in patients undergoing elective surgery.^[55]^ The recommended glucose target for the perioperative period is 80 to 180 mg/dL (4.4–10 mmol/L), while avoiding hypoglycemia.^[56,57]^ For diabetic patients undergoing surgery, a basal-bolus insulin regimen (i.e., a basal long-acting insulin given before bed and additional short-acting insulin doses before meals) is recommended over a sliding-scale regimen (i.e., only short-acting insulin before meals) as the basal-bolus regimen closely resembles the body’s normal insulin release and is associated with better glycemic control and lower postoperative complications.^[58,59]^ For patients who cannot tolerate an oral diet, basal insulin together with correction insulin based on their blood glucose level is advised. Pre-meal and bedtime capillary blood glucose levels should be measured to guide therapy.^[60]^ Meta-analyses of RCTs among patients undergoing surgery showed that intensive glucose control increased the risk of hypoglycemia, with no impact on length of stay compared to conventional glucose control.^[57,61]^ When the patient receives glucocorticoids in the perioperative period, the duration of action of the steroid should be limited to prevent hyperglycemia (Table 7).

**Table 7:** Preoperative diabetes monitoring recommendations and implementation strategies.

	GRADE
Preoperative recommendation	
Diabetes monitoring with HbA1c and glucose levels in high-risk patients is recommended	Quality of evidence: Moderate Recommendation Grade: Strong
Preoperative implementation strategy	
All patients with a glucose level over 120 mg/dL will have an HbA1c drawn unless one has been performed within the last 3 mo. Consider delaying elective surgery if HbA1c is above 8–9% (68–75 mmol/mol). Refer patient back to Primary Care Physician or Endocrinologist for more aggressive management of diabetes	

HbA1c = hemoglobin A1C.

#### 3.1.6. Preoperative computed tomography angiography of aorta and iliac vessels.

ALIF surgery involves mobilization of the major blood vessels in the abdomen, mainly the aorta, the inferior vena cava, and the iliac vessels. This increases the risk of inadvertent vascular injury including bleeding, thrombosis, and dissection. One retrospective review showed the incidence of vascular injury in patients undergoing ALIF to be 7.8%.^[17]^ Patients with a history of peripheral arterial disease (PAD) are at high risk of thrombosis and plaque embolization while undergoing ALIF surgery. Risk factors for PAD include age, history of stroke, smoking, diabetes, hypertension, ischemic heart disease, and hyperlipidemia.^[62,63]^ A multicenter study in Japan showed a PAD prevalence of 6.7% in patients with lumbar spinal stenosis.^[63]^ Severe intermittent claudication is a typical symptom of both PAD and lumbar spinal stenosis. As a result, PAD may be undiagnosed in patients presenting for lumbar fusion surgery. A prospective cohort study among ALIF patients showed preoperative computed tomography angiography (CTA) influenced surgical decision making in 21% of patients, and atherosclerotic disease was detected in 17% of patients.^[64]^ In 1 retrospective study, male patients with diabetes and/or hypertension were recommended to undergo a routine CTA when being evaluated for lumbar spinal stenosis.^[65]^ Therefore, preoperative CTA of the aorta and iliac vessels is recommended to detect atherosclerotic disease and to evaluate prevertebral vascular anatomy in high-risk patients.^[65]^ In those patients presenting with severe atherosclerotic disease or prior stenting on CTA of the aorta and iliac vessels, an ALIF is not recommended and an alternative spinal procedure should be considered (Table 8).

**Table 8:** Preoperative computed tomography angiography of aorta and iliac vessels recommendations and implementation strategies.

	GRADE
Preoperative recommendation	
Preoperative CTA of aorta and iliac vessels is recommended in patients with a history of stroke, PAD, or smoking	Quality of evidence: Moderate Recommendation Grade: Strong
Preoperative implementation strategy	
Order preoperative CTA of the aorta and iliac vessels in patients with a history of stroke, PAD, or smoking once surgery has been offered.	

CTA = computed tomography angiography, PAD = peripheral artery disease.

#### 3.1.7. Preoperative fasting and oral carbohydrate load.

Restriction of food and fluid intake prior to general anesthesia has been practiced for a long time to reduce the risk of gastric content regurgitation. After induction of anesthesia, the gag, cough, and swallowing reflexes are depressed, increasing the risk for pulmonary aspiration which can lead to pneumonia, and in rare cases, death.^[66]^ Fasting before surgery can reduce this risk by decreasing the volume and acidity of the gastric content.^[67]^ However, prolonged preoperative fasting can deprive patients of nutrition and hydration. In a Cochrane Review of 6 RCTs, the rate of aspiration and the volume and pH of gastric content among fasting patients versus reduced-fasting patients was comparable.^[66]^ The European Society of Anesthesiology and the American Society of Anesthesiology both recommend that clear liquids (i.e., water, black coffee, or tea) can be ingested up to 2 hours before induction of anesthesia and a light solid meal can be ingested up to 6 hours before induction of anesthesia.^[68,69]^

Surgery sparks a cascade of metabolic, endocrine, and hemodynamic responses in the body which can contribute to insulin resistance, protein breakdown, and gluconeogenesis, resulting in hyperglycemia and a negative nitrogen balance. Hence, a preoperative oral carbohydrate load is recommended in patients undergoing surgery, except in those with uncontrolled diabetes.^[70]^ Although RCTs of spine surgery patients could not prove the benefits of an oral carbohydrate load,^[71,72]^ the literature on colorectal and vascular surgeries showed that patients given an oral carbohydrate load shortly before surgery have improved postoperative insulin sensitivity when compared to those with just an overnight fast.^[73,74]^ Preoperative oral carbohydrate load has also been correlated to an earlier return of gut function and a significant reduction in the hospital length of stay.^[75]^ Our strategy will be to give 50 g of clear liquid oral complex carbohydrates (i.e., maltodextrin) with low osmolality (~230 mOsm/kg H_2_O) patients 2 hours before induction of anesthesia.^[73,76]^ For best results, the dose should be consumed within 5 to 10 minutes to enhance insulin secretion (Table 9).^[76]^

**Table 9:** Preoperative fasting and oral carbohydrate load recommendations and implementation strategies.

	GRADE
Preoperative recommendation #1	
Preoperative reduced fasting is recommended	Quality of evidence: High Recommendation Grade: Strong
Preoperative implementation strategy #1	
Clear liquids can be permitted for up to 2 h and light solid meal for up to 6 h before induction of anesthesia	
Preoperative recommendation #2	
Preoperative administration of oral carbohydrate load before induction of anesthesia may be considered	Quality of evidence: Moderate Recommendation Grade: Strong
Preoperative implementation strategy #2	
Patients are recommended to drink a formula with low osmolality and complex carbohydrates (i.e., Ensure Pre-Surgery clear [50 g carbohydrates]) 2 h before induction of anesthesia. For best results, the dose before surgery should be consumed within 5–10 min to enhance insulin secretion	

#### 3.1.8. Prevention of postoperative nausea and vomiting.

Postoperative nausea and vomiting (PONV) is common in patients undergoing surgery with a general incidence of 30% and an incidence of 80% in high-risk patients.^[77]^ Severe PONV may result in dehydration, delayed return of oral intake, prolonged length of stay, and increased healthcare costs. It is also a leading cause of patient dissatisfaction.^[78]^ A prior history of PONV, the female sex, a history of motion sickness, age, the duration and type of surgery, the type of anesthetics, and pain scores are independent predictors of PONV.^[79]^ RCTs have shown that a multimodal antiemetic approach during the preoperative, intraoperative, and postoperative periods can considerably reduce the incidence of PONV in high-risk patients and is also associated with higher patient satisfaction.^[80,81]^ Preoperative assessment instruments like Apfel’s simplification score or Koivuranta score can be used to stratify a patient’s risk for PONV^[77]^ and guide a prophylactic antiemetic regimen.

Commonly used pharmacologic antiemetics for prophylaxis in the perioperative period in adults include 5-HT3 receptor antagonists (i.e., ondansetron), NK-1 receptor antagonists (i.e., aprepitant), corticosteroids (i.e., dexamethasone), antihistamines (i.e., dimenhydrinate and diphenhydramine, dopamine antagonists (i.e., droperidol or haloperidol), and anticholinergics (i.e., transdermal scopolamine).^[77]^ Combination therapy for PONV prophylaxis is preferable to single drug therapy due to the additive effects of antiemetics acting on different receptors, thereby increasing their efficacy.^[77]^ In high-risk patients, a scopolamine patch is often given in preoperative holding the day of surgery (POD#0).^[77]^ Aprepitant may be used as an alternative. During surgery, corticosteroids are generally given to decrease the risk of PONV.^[77]^ In addition, ondansetron may be added intraoperatively to further suppress PONV. In the post-anesthesia care unit, some high-risk patients can still develop PONV despite receiving prophylactic antiemetics. They can be given rescue antiemetics from a drug class different to the ones administered pre- and intraoperatively.^[77]^ Dopamine antagonists (e.g., droperidol) and antihistamines (e.g., promethazine) can be useful in these cases. Adverse effects can be limited by using the lowest recommended dose of a drug whenever possible.^[82]^

Intraoperatively, the use of volatile anesthetic agents, nitrous oxide, and intravenous (IV) opioids increases the risk of PONV. An intraoperative multimodal approach to reduce PONV includes avoiding volatile anesthetics, using IV propofol for induction and maintenance of anesthesia, reducing intraoperative use of opioids, and avoiding fluid overload.^[77]^ Bariatric surgery patients who received opioid-free total IV anesthesia experienced significantly lower rates and severity of PONV compared to patients who received volatile-opioid anesthesia despite both groups receiving similar triple PONV prophylaxis.^[83,84]^ PONV can also be reduced by combining general and regional anesthesia, which in turn reduces perioperative opioid consumption.^[85,86]^

Postoperatively, glucocorticoids should be continued on all patients for up to 24 hours (unless contraindicated) due to their robust anti-inflammatory and antiemetic properties.^[87]^ Ondansetron should be considered as a first-line antiemetic for PONV due to its high efficacy and safety profile.^[77]^ In recalcitrant cases of PONV, rescue antiemetics from a different drug class should be given as discussed above.^[77]^ Once rescue therapy is initiated, the patient should be evaluated for any inciting medication or mechanical factors that could be contributing to PONV (e.g., opioid patient-controlled analgesia, abdominal obstruction, blood in pharynx) (Table 10).^[82]^

**Table 10:** Preoperative prevention of postoperative nausea and vomiting recommendations and implementation strategies.

	GRADE
Preoperative recommendation	
Preoperative risk assessment for PONV and administration of prophylactic antiemetic regimen based on risks is recommended	Quality of evidence: High Recommendation Grade: Strong
Preoperative implementation strategy	
In high-risk patients consider giving a scopolamine patch or aprepitant in the preoperative holding area prior to surgery	
Intraoperative recommendation	
A multimodal approach using TIVA while limiting volatile anesthetics and opioids where possible is recommended. Give corticosteroids and an antiemetic during surgery to prevent PONV	Quality of evidence: High Recommendation Grade: Strong
Intraoperative implementation strategy	
Recommend giving dexamethasone 10 mg IV following induction and ondansetron 4 mg IV immediately prior to emergence. Minimize volatile anesthetics where possible, using propofol for induction and maintenance of anesthesia (i.e., TIVA). Minimize intraoperative use of opioids by using opioid-sparing analgesics (e.g., magnesium sulfate MgSO_4_ and ketamine) and combining regional anesthesia. Avoid fluid overload	
Postoperative recommendation	
Postoperative rescue with a different class of antiemetic for patients with persistent PONV is recommended	Quality of evidence: High Recommendation Grade: Strong
Postoperative implementation strategy	
Consider giving a dopamine antagonist or an antihistamine in the PACU for rescue of PONV. Antiemetics in the immediate postoperative period should be of a different receptor class than those given preoperatively or intraoperatively. Once the patient is transferred to the floor from the PACU, recommend redosing corticosteroids (up to 24 hours) and ondansetron if PONV persists	

MgSO_4_ = magnesium sulfate, PACU = post anesthesia care unit, PONV = postoperative nausea and vomiting, TIVA = total intravenous anesthesia.

#### 3.1.9. Antimicrobial prophylaxis.

Surgical site infections (SSI) constitute a large proportion (31%) of hospital acquired infections.^[88]^ SSI after spine surgery is associated with increased hospital costs, morbidity, and mortality.^[88]^ The incidence of SSI after spine surgery varies from 0.7% to 16%.^[89]^ Though there are no universally accepted guidelines for antimicrobial prophylaxis in spinal fusion, a review in the spine surgery literature shows that preoperative decolonization of methicillin-sensitive and methicillin-resistant *Staphylococcus aureus* reduces SSI in patients.^[90]^ Patients are decolonized by treating with intranasal 2% mupirocin ointment and 2% chlorhexidine gluconate (CHG) showers for 5 days before surgery.^[90]^ This procedure has been substantiated by RCTs in patients undergoing orthopedic procedures.^[91–93]^ However, CHG showers can cause skin irritation, dryness, and patient discomfort, particularly when used daily. Therefore, further studies evaluating the efficacy of CHG showers for a shorter duration (i.e., the day before surgery) should be carried out since this may improve patient satisfaction and compliance.

Antimicrobial prophylaxis given intravenously prior to skin incision also reduces the risk of SSI. An antibiotic with a short infusion time to be given within 30 minutes prior to skin incision is recommended.^[94]^ A meta-analysis of RCTs in the spine literature showed a significant reduction in SSI in patients receiving intraoperative antibiotic prophylaxis.^[95]^ To reduce the risk of SSI, a broad-spectrum antibiotic covering *S aureus* (e.g., cefazolin) is commonly given 30 minutes before skin incision, with intraoperative re-dosing every 4 hours.^[94,96]^ Patients with a history of methicillin-resistant *S aureus* infection are given vancomycin 15 mg/kg IV prior to skin incision and for 24 hours after surgery.^[90]^ In high-risk patients, such as those undergoing instrumented or complicated spine surgery, antimicrobial prophylaxis regimens with expanded gram-negative bacteria coverage and the addition of vancomycin or gentamycin powder within the surgical site may be considered.^[97]^

Intraoperative skin preparation with a topical antiseptic can reduce the burden of the skin flora and in turn reduce the incidence of SSI.^[98]^ The most commonly used intraoperative skin preparation agents include CHG, povidone-iodine, and isopropyl alcohol.^[98]^ However, the ideal skin preparation regimen in spine surgery remains unclear. A meta-analysis of RCTs among patients undergoing various surgeries suggests that alcohol-based agents are superior to aqueous solutions when combined with either chlorhexidine or povidone-iodine.^[98,99]^ It also demonstrated that bacteria on the skin were significantly reduced by allowing the povidone-iodine preparation to dry for 10 minutes before incision Table 11).^[100]^

**Table 11:** Antimicrobial prophylaxis recommendations and implementation strategies.

	GRADE
Preoperative recommendation	
Preoperative decolonization of methicillin-sensitive and methicillin-resistant *S. aureus* is recommended for all patients	Quality of evidence: Moderate Recommendation Grade: Strong
Preoperative implementation strategy	
Administer preoperative intranasal mupirocin ointment the night before and on POD#0. Recommend chlorhexidine shower using a brush the night before and on POD#0	
Intraoperative recommendation	
Intraoperative broad-spectrum IV antibiotics covering *S. aureus* and skin preparation with an alcohol-based iodine or chlorhexidine solution is recommended	Quality of evidence: High Recommendation Grade: Strong
Intraoperative implementation strategy	
Administer Ancef 30 minutes before skin incision unless contraindicated. Skin preparation with an alcohol-based iodine or chlorhexidine solution is also recommended	

POD = postoperative day

#### 3.1.10. Patient warming.

Patient warming and temperature monitoring guidelines are recommended by the ERAS Society for several types of surgeries, including colorectal, gynecological, and cardiac surgery.^[101]^ It is important to maintain the patient’s core temperature within normothermic range (i.e., 36.0–37.5°C).^[102–104]^ Perioperative core temperature maintenance decreases the risk of hypothermia, which is associated with increased risk of complications including cardiac events (e.g., arrhythmias, myocardial infarction),^[105]^ coagulation disorders with increased bleeding and transfusion requirement,^[106]^ impaired wound healing and wound infection,^[107]^ and pressure ulcers^[108]^ as well as increased length of stay. Hence, patients should have their core temperature monitored continuously, or at least every 10 minutes, during the perioperative period.^[101]^

Preoperative forced air warming for at least 10 to 20 minutes has been shown to considerably reduce the risk of perioperative hypothermia and postoperative shivering.^[108]^ It is aimed at transferring enough heat (200–300 kJ) to prevent post-induction hypothermia without causing any thermal discomfort or sweating.^[109,110]^ The amount of time between the end of pre-warming and the induction of anesthesia should be as brief as possible, ideally less than 10 minutes.^[109]^ All adult patients scheduled for surgery under anesthesia for a duration of more than 30 minutes should be considered for pre-warming.^[101,108]^

After the induction of anesthesia, the core temperature decreases due to the redistribution of heat within the body caused by an anesthesia-mediated decrease in systemic vascular resistance.^[111,112]^ Therefore, intraoperative active warming is recommended for all patients undergoing ALIF. This can be achieved by using forced air warming blankets and devices, heating mattresses under the patient and circulating water garment systems.^[113]^ Other strategies to prevent hypothermia during surgery includes warming IV fluids and blood products to 37°C using a fluid warming device, and adequately covering the patient (Table 12).^[113]^

**Table 12:** Patient warming recommendations and implementation strategies.

	GRADE
Preoperative recommendation	
Preoperative core temperature monitoring and pre-warming before induction of anesthesia is recommended for all patients undergoing anterior lumbar interbody fusion	Quality of evidence: High Recommendation Grade: Strong
Preoperative implementation strategy	
Preoperative core temperature monitoring and prewarming for at least 10–20 min is recommended before induction of anesthesia. In the event that the patient temperature is <36°C, preoperative efforts will be made to raise their core temp using convection (e.g., Bair Hugger™ system). All patients will be given warmed blankets on arrival	
Intraoperative recommendation	
Intraoperative patient core temperature should be monitored. Normothermia should be maintained through active warming of the patients	Quality of evidence: High Recommendation Grade: Strong
Intraoperative implementation strategy	
Intraoperative patient core temperature should be monitored continuously or at least every 10 min. Normothermia should be maintained by active warming of the patients by using the Bair Hugger™ system and warm blankets. Induction should begin when the patient’s temperature is ≥36°C. In the theater suite, ambient temperature should be at least 21°C when the patient is exposed. Once active warming is started, ambient temperature can be reduced for working conditions. Patients should be adequately covered and exposed only during surgical preparation	

#### 3.1.11. Fluid management.

Intravenous fluid therapy should be aimed at maintaining intravascular volume, cardiac output, and tissue perfusion, while avoiding salt and water overload. Excessive perioperative fluid can lead to an increased length of stay, PONV, and edema of the gut causing prolonged postoperative ileus.^[114]^ Perioperative volume status varies greatly between patients and their unique level of surgical stress response can lead to very different fluid requirements. Preoperatively, liberalized fluid intake should be encouraged up to 2 hours before induction of anesthesia to achieve euvolemia when patients arrive in the operating room. Several RCTs in patients undergoing elective colorectal surgery in an ERAS setting compared routine goal-directed fluid therapy with hemodynamic monitoring to a restrictive fluid regimen.^[115,116]^ They demonstrated that goal-directed fluid therapy showed no clinical benefit over a restrictive fluid regimen for surgeries lasting 2 to 3 hours and requiring an average of 1500 to 2000 mL IV fluids intraoperatively with minimal fluid shift.^[115,116]^

The optimal choice of fluid in the perioperative period is buffered isotonic crystalloids (e.g., Ringer’s lactate or PlasmaLyte).^[117]^ Buffered isotonic crystalloids are considered to be superior to 0.9% normal saline, which causes transient hyperchloremic metabolic acidosis as demonstrated by a RCT of spine surgery patients.^[118]^ Fluid shift can be minimized by avoiding bowel preparation, maintaining hydration with oral fluids up to 2 hours before induction of anesthesia, minimizing bowel-handling during surgery, and avoiding blood loss.

Colloids are human plasma derivatives (i.e., human albumin, fresh frozen plasma) or semisynthetic preparations (i.e., gelatins, hydroxyethyl starches) composed of larger particles that stay in the circulation longer than crystalloids.^[119]^ They are often given in conjunction with crystalloids to avoid fluid overload. RCTs comparing crystalloids alone versus crystalloids in combination with colloids in patients undergoing major surgeries have demonstrated lower IV fluid volumes with colloid-based resuscitation; the differences in clinical outcomes between the 2 groups were not significant.^[120,121]^ A RCT of patients undergoing major abdominal surgery demonstrated reduced postoperative complications when colloids were used with buffered crystalloids.^[122]^ Nevertheless, further studies are warranted to recommend the routine use of colloids in perioperative fluid management.^[123]^

Postoperative fluid management also plays a key role in ensuring adequate perfusion and stable hemodynamics.^[124]^ IV fluids should be restricted postoperatively once the patient starts oral nutrition, unless clinically indicated. Excess IV crystalloids after surgery have been associated with hypoproteinemia and postop ileus.^[124,125]^ Therefore, eating and drinking should be encouraged soon after surgery as it reduces the risk of infection, improves patient satisfaction, and reduces hospital stay.^[126]^ Postoperative oliguria, a normal neurohormonal response to surgical stress,^[127]^ is often the result of fluid retention. Thus, IV fluid infusion should not be given as a first-line treatment. If clinically indicated, a thorough workup to determine the etiology of postoperative oliguria should be considered to guide proper management (Table 13).

**Table 13:** Fluid management recommendations and implementation strategies.

	GRADE
Preoperative recommendation	
Recommend liberalized fluid intake up to 2 h before induction of anesthesia to achieve euvolemia	Quality of evidence: High Recommendation Grade: Strong
Preoperative implementation strategies	
Patients are recommended to drink clear fluids up to 2 h before induction of anesthesia, including a complex oral carbohydrate drink (i.e., Ensure Pre-Surgery clear [50 g carbohydrates])	
Intraoperative recommendation	
Intraoperative IV fluid therapy using buffered isotonic crystalloids is recommended to maintain euvolemia in all patients undergoing ALIF	Quality of evidence: Moderate Recommendation Grade: Strong
Intraoperative implementation strategies	
Recommend use of buffered isotonic crystalloids (i.e., Ringer’s lactate or PlasmaLyte) instead of 0.9% normal saline for hypovolemia treatment	
Postoperative recommendation	
Postoperative IV fluids should be restricted once the patient starts oral intake, unless clinically indicated	Quality of evidence: Moderate Recommendation Grade: Strong
Postoperative implementation strategy	
Saline-lock IV once patient has successfully tolerated 300cc oral intake of a clear liquid diet (Ensure Clear)	

ALIF = anterior lumbar interbody fusion

#### 3.1.12. Management of urinary catheters.

Urinary catheters are placed intraoperatively to monitor fluid balance and prevent bladder distention. However, it is well established that prolonged catheterization can cause urinary tract infections.^[128]^ Preoperatively, risk factors for catheter-associated urinary tract infections (CAUTI) following ALIF have been found to include age ≥ 60 years, female sex, alcohol use, steroid use, and open wound or wound infections.^[129]^ Additionally, ALIF research has shown that operative time in patients with CAUTI following ALIF had significantly longer operative times.^[130]^ Regardless, removal of indwelling urinary catheters as soon as possible is absolutely key in preventing CAUTIs.^[129]^

Prolonged catheterization can also cause postoperative urinary retention (POUR), which can lead to sepsis and increased length of hospital stay.^[131]^ The risk of developing POUR after lumbar spine surgery is approximately 5% and is most commonly associated with the male sex, benign prostatic hyperplasia, age, diabetes, and depression.^[132]^ Limiting the use of urinary catheters in spine surgery patients can reduce these adverse events and promote early patient ambulation.^[133]^ Catheterization should be avoided in patients undergoing short elective spinal procedures and when used, should be removed within hours of surgery. Post-void volumes should be measured to monitor for POUR (Table 14).^[134]^

**Table 14:** Management of urinary catheters recommendations and implementation strategies.

	GRADE
Intraoperative/postoperative recommendation	
Urinary catheters when used should be removed postoperatively as soon as clinically indicated	Quality of evidence: Moderate Recommendation Grade: Strong
Intraoperative/postoperative implementation strategy	
Urinary catheters should be avoided in short elective procedures, and when used should be removed as soon as possible after surgery	

#### 3.1.13. Surgical approaches.

The retroperitoneal approach is the preferred approach for accessing the anterior lumbar spine due to its superior safety profile compared to the transperitoneal approach. Complications traditionally associated with the transperitoneal approach,^[18]^ such as an increased risk of retrograde ejaculation,^[18,135]^ paralytic ileus, and injury to the peritoneum and the superior hypogastric plexus are minimized.^[136]^ Additionally, the number of levels than can be accessed with the transperitoneal approach is typically decreased, and requires direct manipulation of intra-abdominal contents, which could increase the risk of ileus or injury to the intraperitoneal structures.^[137]^

Minimally invasive surgery is an independent predictor of reduced complications after colorectal surgery.^[138]^ In spine surgery, it reduces postoperative pain, opioid requirement, and blood loss as well as promotes early mobilization.^[139,140]^ Specific to ALIF surgery, the incidence of postoperative ileus in the less invasive mini-open ALIF approach is less than that in open ALIF.^[141–143]^ Therefore, mini-open ALIF should be considered, when possible, given the surgeon’s experience, the surgery goals, and its availability at the local institution (Table 15).^[140,144]^

**Table 15:** Surgical approaches recommendations and implementation strategies.

	GRADE
Intraoperative recommendation	
The retroperitoneal approach is recommended for anterior lumbar interbody fusion surgeries	Quality of evidence: Low Recommendation Grade: Weak
Intraoperative implementation strategy	
The retroperitoneal approach should be used, and when feasible, incorporate minimally invasive techniques	

#### 3.1.14. Intraoperative neurophysiological monitoring.

Intraoperative neurophysiological monitoring is employed in spine surgery to avoid neurological complications.^[145]^ The most commonly used modalities include somatosensory evoked potentials (SSEP), electromyography, and motor evoked potentials. In lumbar spine surgery, a combination of SSEPs (high specificity) and electromyography (high sensitivity) is considered to be the best modalities to prevent nerve root injuries.^[146^ Studies have shown that most patients undergoing ALIF at the L4-L5 level are subject to compression of the left iliac vessels, causing transient desaturation of the left lower extremity. This vascular compromise correlates with changes in SSEP signals, which may indicate a left iliac artery thrombosis.^[147]^ Several case reports have demonstrated that SSEP combined with pulse oximetry monitoring of the left great toe is highly valuable in detecting left iliac artery occlusion in anterior spinal procedures.^[148–151]^ Baseline neurophysiological recordings should be obtained after induction of anesthesia but prior to incision to compare changes in signals during surgery (Table 16).^[152]^

**Table 16:** Intraoperative neurophysiological monitoring recommendations and implementation strategies.

	GRADE
Intraoperative recommendation	
Intraoperative neurophysiological monitoring is recommended for all patients	Quality of evidence: Moderate Recommendation Grade: Strong
Intraoperative implementation strategy	
Routine intraoperative neurophysiological monitoring, including somatosensory-evoked potentials and electromyography, is recommended for all patients undergoing anterior lumbar interbody fusion surgery	

#### 3.1.15. Intraoperative pulse oximetry of the left great toe.

Vascular adverse events such as arterial thrombosis, embolus, dissection, and vascular laceration during retraction of the vessels are well-described during anterior lumbar surgeries. Therefore, it is important to detect and treat them promptly to minimize limb ischemia and prevent limb loss.^[153]^ Extensive mobilization of the left iliac vessels and the aorta is often required for adequate exposure during the retroperitoneal approach of ALIF. These vessels may experience significant stretch and compression.^[147]^ Therefore, pulse oximetry of the left great toe should be considered to monitor blood flow through the left lower extremity. Prospective studies and case reports have shown that continuous pulse oximetry of the left great toe alone or combined with neurophysiologic monitoring like SSEP during surgery can help in the timely detection of limb ischemia.^[147,154,155]^ In addition, the intermittent release of the retractors on the major vessels has been shown to reduce the incidence of vessel complications during ALIF (Table 17).^[156]^

**Table 17:** Intraoperative pulse oximetry of the left great toe recommendations and implementation strategies.

	GRADE
Intraoperative recommendation	
Intraoperative pulse oximetry of the left great toe is recommended during anterior lumbar interbody fusion procedures	Quality of evidence: Moderate Recommendation Grade: Strong
Intraoperative implementation strategy	
See above	

#### 3.1.16. Intraoperative mean arterial pressure monitoring.

Mean arterial pressure (MAP) is closely monitored during surgery to ensure patient safety by promptly correcting changes in blood pressure. Several traumatic spinal cord injury studies recommend a MAP goal of 80 to 85 mm Hg.^[157–160]^ However, one prospective study of patients undergoing elective lumbar spine surgery demonstrated that a MAP range of 70 to 100 mm Hg leads to improved outcomes and fewer complications.^[161]^ Another prospective study showed that raising MAP above 85 mm Hg in response to changes in intraoperative neurophysiological monitoring signals can successfully restore patients back to baseline in 20% of cases (Table 18).^[162]^

**Table 18:** Intraoperative mean arterial pressure monitoring recommendations and implementation strategies.

	GRADE
Intraoperative recommendation	
Intraoperative mean arterial pressure should be maintained at a range of 70–100 mm Hg is recommended	Quality of evidence: Low Recommendation Grade: Weak
Intraoperative implementation strategy	
See above	

#### 3.1.17. Prophylaxis against venous thromboembolism.

The incidence of symptomatic DVT and pulmonary embolism after elective spine surgery is 0.66% and 0.88%, respectively.^[163]^ The intraoperative mobilization of the vessels during an ALIF can increase the risk of DVT from 0.7% to 5%.^[15]^ Therefore, postoperative mechanical and chemoprophylaxis should be considered for all patients undergoing ALIF, especially for those undergoing a combined anterior-posterior procedure.^[164]^ Patients are also encouraged to ambulate early after surgery because it reduces the risk and incidence of venous thromboembolism (VTE) in the immediate postoperative period.^[165,166]^ Prophylaxis for VTE includes both mechanical prophylaxis (i.e., compression stockings, intermittent pneumatic compression devices) and chemoprophylaxis (i.e., low molecular weight heparin, unfractionated heparin, fondaparinux, rivaroxaban). Mechanical prophylaxis provides circumferential compression to the patient’s legs, preventing venous stasis and improving blood flow whereas chemoprophylaxis acts pharmacologically to hinder the clotting cascade.

Currently, there are no official guidelines recommending the timing of initiation and the duration of chemoprophylaxis in spine surgery patients.^[167]^ Chakravarthy et al’s^[168]^ ERAS protocol for spine surgery patients recommends mechanical prophylaxis immediately after surgery, followed by chemoprophylaxis with 5000 IU of unfractionated heparin twice daily on postoperative day two, unless contraindicated. This is supported by a similar regimen in a retrospective cohort study, which demonstrated a decrease in the incidence of VTE without increased morbidity in spine surgery patients.^[169]^ According to a retrospective review, starting low molecular weight heparin 24 to 36 hours after spine surgery is safe.^[170]^ Given their efficacy, low cost, and low complication rates,^[171–173]^ both mechanical prophylaxis and chemoprophylaxis is recommended postoperatively in ALIF patients due to their increased risk of developing a VTE (Table 19).

**Table 19:** Prophylaxis against venous thromboembolism recommendations and implementation strategies.

	GRADE
Postoperative recommendation	
Routine use of mechanical and chemoprophylaxis against venous thromboembolism is recommended for all patients	Quality of evidence: Moderate Recommendation Grade: Strong
Postoperative implementation strategy	
Recommend mechanical prophylaxis using compression stockings and intermittent pneumatic compression devices for venous thromboembolism in all patients undergoing ALIF. Chemoprophylaxis such as subcutaneous low molecular weight heparin or unfractionated heparin should be started within 24 h after surgery in all ALIF patients, unless contraindicated	

ALIF = anterior lumbar interbody fusion.

#### 3.1.18. Nutrition.

Perioperative malnutrition is a known independent predictor of poor postoperative outcomes, including increased hospital length of stay, readmissions, morbidity, and mortality.^[174]^ Malnutrition is also considered to increase the risk of SSI by impairing wound healing and prolonging inflammation. With an aging population and patients presenting with an increasing array of comorbidities, perioperative nutritional status should be optimized to achieve the best surgical outcome.^[175]^ The diagnosis of preoperative malnutrition is ascertained by using a combination of standardized nutritional screening tools (e.g., Malnutrition Screening Tool^[176]^), anthropometric measurements (i.e., calf or arm muscle circumference, triceps skinfold), and laboratory testing (i.e., serum albumin, prealbumin, total lymphocyte count).^[175]^ The American Society for ERAS and Perioperative Quality Initiative Joint Consensus recommends that patients at high risk of malnutrition according to an initial nutritional screening should be referred to a registered dietician for a complete nutritional assessment and intervention.^[177]^ Treatment includes meal fortification with protein and a combination of essential nutrients (i.e., immunonutrition) like arginine, glutamine, omega-3-fatty acids, and nucleotides for at least 7 days before surgery.^[177]^ Elective lumbar spine surgery patients that received a nutritional regimen with protein supplementation had lower length of stay, reduced incidence of electrolyte disturbances, and higher postoperative albumin levels on postoperative day three (POD#3) compared to a control group.^[178]^ Furthermore, colorectal surgery patients that received preoperative protein optimization (i.e., 32 g protein/d) and immunonutrition supplements starting 7 days before surgery through postoperative day five exhibited fewer postoperative complications.^[179]^

A period of starvation is a common practice after many elective surgeries. The stomach is often decompressed with a nasogastric tube during surgery while IV fluids are given, with oral feeding being introduced postoperatively as gastric dysmotility resolves.^[180]^ The rationale for this is to prevent nausea and vomiting. As bowel motility returns (i.e., bowel sounds, flatus, bowel movement), patients are given a clear liquid diet and slowly advanced to a regular diet.^[180]^ However, RCTs involving patients undergoing various surgeries concluded that there is no advantage in withholding fluids and solid food past the immediate postoperative period.^[180–182]^ Indeed, patients that received early oral feeding demonstrated a quicker recovery of bowel function, a decrease in septic complications, a reduced length of stay, and improved patient satisfaction.^[180,182]^ For these reasons, the American Society for ERAS Joint Consensus Statement recommends early oral nutrition to reduce postoperative complications and improve surgical outcomes.^[177]^ In most studies, patients were started on oral clear liquids as soon as tolerated^[181]^ or within 24 hours of surgery followed by advancing to a solid diet as tolerated by the patients.^[182–184]^

The exact composition of the optimal postoperative diet is still under debate. Nevertheless, a high-protein and immunonutrition diet is very important.^[179,185,186]^ Studies have shown that early introduction of a high-protein diet in patients undergoing colorectal surgery or trauma surgery can significantly reduce length of stay, morbidity, and mortality rates.^[187–189]^ Similarly, immunonutrition supplements reduced postoperative complications in colorectal surgery patients.^[179]^ Hence, we believe the postoperative diet of all ALIF patients should be supplemented with protein and immunonutrition. Patients can be started soon after surgery on a clear liquid high in protein and essential amino acids (e.g., Juven nutrition powder or Ensure Clear) followed by protein shakes twice daily for 5 days after surgery (Table 20).^[179]^

**Table 20:** Nutrition recommendations and implementation strategies.

	GRADE
Preoperative recommendation	
Preoperative nutritional screening and optimization of nutritional status is recommended for all patients undergoing anterior lumbar interbody fusion.	Quality of evidence: Moderate Recommendation Grade: Strong
Preoperative implementation strategy	
All patients will have a nutritional screening. Malnourished patients will be referred to a nutritionist for further consultation. All patients will receive protein shake supplements (i.e., Ensure Surgery) twice daily for at least 5 d before surgery	
Postoperative recommendation #1	
Recommend early oral clear liquid diet and advance as tolerated in the absence of postoperative nausea and vomiting for all patients	Quality of evidence: High Recommendation Grade: Strong
Postoperative implementation strategy #1	
Initiate a protein-rich clear liquid diet (e.g., Ensure Clear) as soon as possible after surgery and advance diet as tolerated in the absence of postoperative nausea and vomiting	
Postoperative recommendation #2	
Early postoperative high-protein diet is recommended	Quality of evidence: High Recommendation Grade: Strong
Postoperative implementation strategy #2	
Protein shakes (e.g., Ensure Surgery) are recommended twice daily for at least 5 d postoperatively in conjunction with meals	

#### 3.1.19. Early mobilization.

The traditional practice of bed rest after surgery has been associated with negative outcomes due to increased risk of thromboembolism, pneumonia, muscle wasting, and physical deconditioning.^[165]^ Hence, all patients are encouraged to mobilize as soon as they are able to after surgery.^[190]^ An RCT and a systemic review of patients undergoing elective spine surgery showed that early mobilization on the POD#0 could be safely initiated,^[191,192]^ and combining it with a prehabilitation program significantly shortened the length of stay and improved the functional status of patients.^[193]^ Another study revealed that early ambulation after spine surgery significantly reduced the incidence of postoperative complications such as pneumonia and DVT.^[191,194]^ Consensus-based best practice guidelines for adolescents undergoing posterior spine surgery recommend sitting on the edge of the bed on POD#0 and ambulation on postoperative day one (POD#1).^[195]^ Although no guidelines are currently available for early mobilization in adults undergoing anterior spine surgery, findings from a cohort study suggests that ambulation on POD#0 is both safe and effective in reducing postoperative complications.^[195–197]^ In order to optimize daily mobilization goals in the postoperative period, a personalized care plan should be made preoperatively to set clear expectations for patients undergoing surgery.^[198]^ In addition, adequate pain control should be maintained while drains and urinary catheters should be removed as soon as possible to promote early mobilization (Table 21).^[199]^

**Table 21:** Early mobilization recommendations and implementation strategies.

	GRADE
Postoperative recommendation	
Early mobilization is recommended for all patients	Quality of evidence: Moderate Recommendation Grade: Strong
Postoperative implementation strategy	
Early mobilization should be achieved by involving a multidisciplinary team. This includes sitting on the edge of the bed or chair and ambulation with assistance on POD#0	

POD = postoperative day.

#### 3.1.20. Rehabilitation.

Postoperative rehabilitation is aimed at restoring muscle strength and mobility of patients during the recovery period after a major surgery. It involves a multidisciplinary team including physical therapists and occupational therapists to help patients promptly return to their activities of daily living (ADL).^[200]^ Rehabilitation involves cardiovascular exercise (i.e., walking), soft tissue mobilization (i.e., massage therapy), motor control and strengthening (i.e., proper strengthening of the trunk extensors and balancing core muscles), joint mobilization (i.e., proper posture, functional mobility, decreasing stress on the healing fusion) and patient education.^[200]^ A RCT involving patients undergoing elective spine surgery showed that prehabilitation combined with postoperative acute care (i.e., hospital-based) physical therapy (PT) improves patient outcomes and reduces hospital length of stay.^[193]^ Acute care PT after surgery is recommended twice daily from POD#1 until discharge from the hospital.^[193,196]^

Cochrane Reviews of spine surgery literature revealed that outpatient PT beginning as early as 4 to 6 weeks after surgery and at least 3 to 4 times per week for 6 to 12 weeks leads to a more rapid decrease in pain and disability.^[201–203]^ Another review concluded that outpatient PT improves both the short- and long-term functional status of patients.^[204]^ Although there is no consensus on the exact protocol for outpatient spine surgery rehabilitation, PT can be safely initiated at 4 to 6 weeks after surgery.^[201–203]^

Immediate postoperative hospital-based occupational therapy (OT) also plays an important role in rehabilitation for elective spinal fusion surgery patients.^[205]^ OT provides instructions to help the patients perform ADL (i.e., bathing, eating, using the bathroom, and dressing), proper ergonomics, and posture at work. A RCT involving orthopedic surgery patients demonstrated that acute care OT improves the patient’s ability to return to independent living at home.^[206]^ Training with the occupational therapists encourages and motivates the patients to perform everyday activities shortly after surgery.

Inpatient rehabilitation with PT and OT should be considered for patients with transfer and ADL functional dependencies (i.e., moderate assist or worse).^[207]^ Several cohort studies in patients undergoing elective orthopedic surgery demonstrated that inpatient rehabilitation improved functional outcomes after surgery.^[208–210]^ However, there is limited evidence on the role of inpatient rehabilitation in improving the clinical outcomes after spinal fusion.^[211]^

Home health may be beneficial for patients requiring supervision-assist to minimal-assist. Home healthcare nurses visit the patient’s home to assess their needs and facilitate their independence at home.^[212]^ This is achieved by patient education (i.e., a patient guidebook), patient advocacy (i.e., psychosocial support), spiritual and aesthetic communication (i.e., weekly counseling and emotional support), and case management (i.e., individual care plan and assessment). Home healthcare services can improve ADL and reduce psychosocial distress after surgery in elective spine surgery patients (Table 22).^[212]^

**Table 22:** Rehabilitation recommendations and implementation strategies.

	GRADE
Postoperative recommendation	
Recommend acute care physical therapy and occupational therapy during hospitalization for all patients. Recommend outpatient PT for all patients	Quality of evidence: High Recommendation Grade: Strong
Postoperative implementation strategy	
Acute care PT and OT is recommended during hospitalization for all ALIF patients beginning on POD#1. Outpatient physical therapy is recommended for all ALIF patients beginning 4–6 wk after surgery for 3–4 times per week. For high-risk patients, consider inpatient rehabilitation and home health	

ALIF = anterior lumbar interbody fusion, OT = occupational therapy, PT = physical therapy.

#### 3.1.21. Lumbar brace.

Lumbosacral orthosis is usually used for the management of low back pain, spondylolisthesis, and spinal deformity. It is also used for restricting the movement of an operated spine.^[213]^ The pain following spine surgery is typically less severe when an external brace is applied due to the increased stability offered by the brace and the increased sense of security that it gives to patients.^[214]^ However, there is no consistent scientific evidence to support the routine use of a postoperative lumbar brace. Recent RCTs involving patients undergoing minimally invasive transforaminal lumbar interbody fusion and posterior spinal fusion concluded that spinal orthosis is not necessary after surgery.^[213,215,216]^ Patients without orthosis had similar fusion rates, pain scores, and disability index scores. Moreover, eliminating back braces altogether resulted in a more comfortable postoperative period, an easier recovery, and higher patient satisfaction. Nevertheless, bracing is necessary for patients at increased risk for nonunion or pseudo-arthrosis, including smokers, diabetics, and those that have undergone multiple-level ALIF (Table 23).^[217]^

**Table 23:** Lumbar brace recommendations and implementation strategies.

	GRADE
Postoperative recommendation	
Consider using postoperative lumbar braces when mobilizing anterior lumbar interbody fusion patients	Quality of evidence: Low Recommendation Grade: Weak
Postoperative implementation strategy	
Lumbar brace can be worn when up and ambulating	

#### 3.1.22. Multimodal analgesia.

A standardized perioperative multimodal analgesic protocol improves postoperative outcomes such as pain scores, opioid consumption, and hospital length of stay.^[218]^ This protocol involves combining non-narcotic analgesics like non-steroidal anti-inflammatory drugs (NSAIDs), acetaminophen, gabapentinoids, N-methyl D-aspartate (NMDA) receptor antagonists, and glucocorticoids with opioid analgesics pre- and postoperatively.^[218]^ NSAIDs are opioid-sparing and can reduce PONV by 30%.^[219]^ They can be given safely to patients undergoing elective lumbar spine surgery to reduce pain scores and opioid consumption.^[220,221]^ RCTs involving elective spine surgery patients recommended oral celecoxib at a dose of 200 mg 1 hour before surgery and 200 mg once daily during hospitalization.^[222,223]^ Another RCT in the spine surgery literature demonstrated that ibuprofen can be safely given to patients at a dose of 800 mg IV 30 minutes before surgery without increasing the incidence of adverse effects such as bleeding.^[224]^ An RCT involving orthopedic and abdominal surgery patients recommended postoperative use of ibuprofen at a dose of 800 mg IV or oral every 6 hours to significantly reduce opioid use.^[225]^ Although postoperative complications of NSAIDs include bleeding and pseudoarthrosis, it’s short-term (<2 weeks) perioperative use appears to be safe and does not affect overall spinal fusion rates.^[226]^ Therefore, it is reasonable to give patients a preoperative dose of NSAID, which can be continued up to 2 weeks postoperatively. NSAIDs should be avoided in patients with renal dysfunction, peptic ulcer disease, and known allergy.

Acetaminophen is another important component of the perioperative analgesic regimen. It can be given either as a 1 g oral or 1 g IV dose immediately preoperatively and continued during hospitalization at a dose of 1 g every 6 hours for 48 hours.^[227]^ It functions both as an analgesic and an antipyretic and has an additive effect when combined with NSAIDs and opiates.^[228]^ Acetaminophen should be avoided in patients with hepatic dysfunction and allergy.

Gabapentinoids such as gabapentin and pregabalin are widely used in the treatment of chronic pain and neuropathic pain. Several RCTs conducted in patients undergoing various surgeries demonstrated that gabapentinoids reduced morphine consumption especially during the first 24 to 48 hours after surgery. It also contributed to lower pain scores and movement-related pain.^[229–233]^ The recommended dose of gabapentin is 600 mg given 2 hours before induction of anesthesia in elective lumbar spine surgery patients.^[234]^ Another RCT involving elective spine surgery patients recommended a dose of 600 mg gabapentin 2 hours prior to induction of anesthesia followed by 600 mg given postoperatively at 10 and 22 hours.^[235]^ RCTs involving elective spine surgery patients recommended pregabalin to be given at a dose of 300 mg 1 hour before surgery and continued at a dose of 150 mg twice daily for 2 days after surgery.^[236,237]^

Glucocorticoid steroids (e.g., dexamethasone) are an important class of drugs that are effective in reducing the surgical stress response owing to their strong anti-inflammatory properties.^[238,239]^ A systemic review and meta-analysis of 11 RCTs revealed a reduction in the rate of postoperative complications and hospital stay after a glucocorticoid regimen.^[240]^ Glucocorticoids also decreased the incidence of PONV^[87,241–243]^ and reduced pain scores after elective spine and orthopedic surgery.^[81,244,245]^ Low-dose regimens have been shown to be safe and effective in the postoperative period.^[246]^ Glucocorticoids have many potential adverse effects, including hyperglycemia, infection, and poor wound healing.^[247]^ Therefore, they should be used briefly and should be avoided in poorly controlled diabetics and in immunocompromised patients. Perioperative blood glucose should be monitored in patients with diabetes undergoing ALIF.

NMDA antagonists are another class of opioid-sparing analgesics. Intraoperative IV magnesium sulfate (MgSO_4_) acts as a noncompetitive antagonist of the NMDA receptor, which makes it a useful adjuvant for perioperative analgesia in patients undergoing spine surgery.^[248,249]^ A RCT involving elective spine surgery patients demonstrated that intraoperative 50 mg/kg IV MgSO_4_ given as a single bolus dose significantly reduced opioid consumption, improved patient discomfort, and reduced the incidence of PONV.^[248,249]^ Intraoperative low-dose ketamine combined with MgSO_4_ reduced opioid use and improved pain scores at 48 hours postoperatively in elective spine surgery patients.^[250]^ Ketamine, another NMDA antagonist, reduces the peri- and postoperative opioid analgesic requirement and prolongs the time to first opioid dose.^[251]^ Low-dose ketamine significantly decreased postoperative morphine consumption and the incidence of PONV in major abdominal and orthopedic surgery patients.^[252–254]^ Methadone can also be effective in providing postoperative pain relief in chronic pain patients. It is peculiar in that it shares the mu-receptor agonist properties of an opioid but exhibits ketamine-like antagonistic properties at the NMDA receptor. Postoperative methadone can improve pain control, reduce opioid requirement, and improve patient satisfaction in elective spinal fusion surgery patients.^[255–257]^

A regional anesthetic block in conjunction with general anesthesia during surgery can minimize the need for postoperative opioid analgesics. It also contributes to a quicker recovery from anesthesia, which in turn can facilitate early oral intake and patient mobilization on POD#0.^[258]^ The ultrasound-guided transverse abdominis plane (TAP) block is simple, safe, and effective for ALIF patients.^[259]^ The ultrasound probe is placed in the mid-clavicular line, midway between the anterior superior iliac spine and subcostal margin to obtain a transverse view of the abdominal muscle layers. Local anesthetic is then injected in the plane between the internal oblique and transverse abdominis muscles to target the nerves passing through them.^[259]^ A TAP block provides motor-sparing analgesia at the site of the abdominal incision, without compromising the postoperative neurological examination.^[259]^ In ERAS, the TAP block has been shown to be superior at reducing hospital length of stay compared to epidural anesthesia.^[260]^ Other regional plane blocks used during spine surgery include ultrasound-guided erector spinae plane block and thoracolumbar interfacial plane block. Posterior spinal fusion patients that received these dorsal spinal blocks reported significantly reduced postoperative pain scores compared to control groups.^[261,262]^ Dorsal spinal blocks can be especially useful for ALIF patients undergoing combined anterior and posterior lumbar procedures.

Muscle relaxants such as methocarbamol, diazepam, and cyclobenzaprine can provide significant symptom relief when used to treat low back pain. The European guidelines for the management of low back pain recommend muscle relaxant only for short-term pain relief due to their adverse effects like drowsiness, dizziness, and risk of dependency.^[263]^ There are currently no studies assessing the effectiveness of muscle relaxants for pain relief after spine surgery.^[264]^ Further research is warranted to make a recommendation on the routine use of muscle relaxants after spine surgery.

Local cryotherapy is a non-pharmacological form of pain management that can be used as an adjunct to medications. It usually involves the application of an ice pack at the surgical wound site. Local cryotherapy reduces edema, hemorrhage, and tissue damage.^[265]^ It also increases pain threshold and tolerance by reducing nerve conduction velocity and decreasing muscle spasm, especially at temperatures less than 27°C.^[265]^ Ice packs are a simple, safe, and cost-effective method of decreasing postoperative pain and opioid requirement in surgery patients (Table 24).^[265–267]^

**Table 24:** Multimodal analgesia recommendations and implementation strategies.

	GRADE
Preoperative recommendation	
A preoperative multimodal opioid-sparing analgesia strategy is recommended	Quality of evidence: High Recommendation Grade: Strong
Preoperative implementation strategy	
Celecoxib 400 mg and acetaminophen 1000 mg by mouth will be administered to patients prior to surgery. Preoperative loading of gabapentin over 5 d will be implemented with a maintenance dose administered immediately before surgery unless the patient is already on a maintenance dose	
Intraoperative recommendation	
Minimize intra-operative use of opioids. Consider combining regional anesthetic blocks with general anesthesia to reduce intra- and postoperative opioid requirements	Quality of evidence: Moderate Recommendation Grade: Strong
Intraoperative implementation strategy	
Minimize intra-operative use of opioids by using ketamine, dexamethasone 10 mg IV, and MgSO_4_ 2 g IV and by employing regional anesthesia where possible. A transverse abdominis plane block is recommended for all patients solely undergoing ALIF surgery. Recommend an erector spinae block for all ALIF patients undergoing an additional posterior stage (i.e., ALIF 360) in conjunction with bilateral rectus sheath blocks or local infiltration of the incision with bupivicaine to cover the ALIF incision anteriorly	
Postoperative recommendation	
Multimodal opioid-sparing analgesic protocol for postop pain management should be used in all patients undergoing ALIF	Quality of evidence: High Recommendation Grade: Strong
Postoperative implementation strategy	
Postoperative pain management using a multimodal opioid-sparing analgesic regimen, including gabapentin, glucocorticoids 4 mg IV every 6 h for 24 h and Ibuprofen IV 800 mg every 8 h for 24 h followed by 800 mg by mouth for 48 h, acetaminophen 1000 mg IV every 6 h for 24 h followed by 1000 mg by mouth every 6 h for 24 h, and muscle relaxers (e.g., methocarbamol). Cryotherapy at the incision site is also recommended. Consider ketamine and methadone to optimize pain control for chronic pain patients	

ALIF = anterior lumbar interbody fusion, IV = intravenous, MgSO_4_ = magnesium sulfate.

#### 3.1.23. Postoperative ileus.

Postoperative ileus is obstipation and intolerance to oral diet due to non-mechanical factors that disrupt the normal propulsive motor activity of the gastrointestinal tract following surgery.^[268]^ Some degree of postoperative ileus is an expected physiological response to abdominal surgery. However, prolonged ileus can lead to patient discomfort, dissatisfaction, prolonged hospitalization, and increased healthcare costs.^[268]^ Unresolved ileus by POD#3 is considered pathological. It can present with symptoms of abdominal pain, bloating, nausea, vomiting, and delayed oral intake.^[269]^ Approximately 7.5% of ALIF patients and 8.4% of combined anterior-posterior procedure patients will experience postoperative ileus.^[270]^ Risk factors for postoperative ileus after elective spine surgery include male gender, African American ethnicity, spinal fusion of >3 levels, increased blood loss, fluid or electrolyte disturbances, and alcohol abuse.^[270]^ Routine use of a perioperative bowel protocol is recommended to facilitate the early return of bowel function after surgery. This protocol includes the use of stool softeners (i.e., docusate) pre- and postoperatively. Although the evidence to support their efficacy in ERAS protocols is weak,^[271]^ a RCT involving major gynecological surgery patients demonstrated that prophylactic stool softeners and laxative reduced the time to first bowel movement after surgery.^[272]^ Therefore, our strategy will be to start oral docusate (Colace) 100 mg once daily starting 5 days before surgery and continue this regimen daily during their hospitalization. Furthermore, laxatives (e.g., bisacodyl) will be given at a dose of 10 mg (oral) twice daily starting on POD#0 and continued until POD#3 to stimulate bowel motility.^[273]^ If constipation is not relieved by the above measure by POD#3, a sodium phosphate (Fleet) rectal enema (19 g monobasic sodium phosphate and 7 g dibasic sodium phosphate/118 mL) once per day will be given.^[274]^

When a patient has no signs of bowel movement, is intolerant to an oral diet by POD#3, and has failed the treatment regimen for constipation, an evaluation for prolonged postoperative ileus should be initiated to rule out mechanical causes of bowel obstruction. In the case of an ileus, a supine and upright abdominal X-ray will often demonstrate dilated small bowels and colon, without a transition zone or free air under the diaphragm.^[275]^ If the X-ray is inconclusive, consider a gastrografin study followed by a surgical consult. Serial abdominal examinations should be performed to monitor the severity of abdominal distention and patient discomfort. A complete blood count and a basic metabolic panel can rule out other causes of ileus such as anemia and electrolyte disturbances (i.e., hypokalemia or hypomagnesemia).^[268]^ Once postoperative ileus is confirmed, treatment usually involves supportive care. This includes removing potential triggers such as opioid analgesics, fluid overload, and electrolyte disturbances. Mobilization is also encouraged.^[276]^ Bowel rest with sips of clear fluids is recommended if tolerated.^[268]^ Nasogastric tube insertion may be considered in patients with severe nausea and vomiting or significant abdominal distention.^[277]^

Sham feeding can also promote recovery of gastrointestinal function. Chewing sugarless gum stimulates the cephalic vagal reflex which in turn promotes intestinal myoelectric activity. It also increases the secretion of gastrin, neurotensin, and pancreatic polypeptides which further enhance gastrointestinal motility.^[278,279]^ Studies have demonstrated that chewing gum is safe and reduces the time to first postoperative bowel movement in major abdominal surgery patients.^[280–283]^ However, there is no evidence to suggest that chewing gum reduces the time to return of bowel function in elective spinal fusion patients.^[284,285]^ Nevertheless, chewing gum may be an acceptable option for all patients given its low cost and low risk profile.^[286]^ Patients may be given chewing gum 3 times a day beginning on POD#0 (Table 25).^[280]^

**Table 25:** Postoperative ileus recommendations and implementation strategies.

	GRADE
Preoperative recommendation #1	
Preoperative routine administration of stool softeners is recommended	Quality of evidence: Moderate Recommendation Grade: Strong
Preoperative implementation strategy	
Administer oral Colace (100 mg) once daily starting 5 d before surgery to reduce the incidence of post-operative ileus	
Postoperative recommendation #1	
Routine use of postoperative stool softeners is recommended.	Quality of evidence: Moderate Recommendation Grade: Strong
Postoperative implementation strategy #1	
Administer oral Colace (100 mg) once daily	
Postoperative recommendation #2	
Routine use of postoperative laxatives is recommended	Quality of evidence: Moderate Recommendation Grade: Strong
Postoperative implementation strategy #2	
Administer Dulcolax (10 mg) twice daily for 3 d. Consider Fleet on POD#3 if patient is constipated	
Postoperative recommendation #3	
Consider performing an ileus workup in patients with no signs of bowel movement, intolerance to oral diet, and/or obstipation despite treatment regimen for constipation	Quality of evidence: Low Recommendation Grade: Weak
Postoperative implementation strategy #3	
Patients with no signs of bowel movement, intolerance to oral diet, and ongoing constipation or obstipation on POD#3 despite bowel protocol should undergo an ileus workup. This includes ruling out mechanical bowel obstruction using serial abdominal X-rays, performing serial abdominal exams, and if indicated, a gastrografin study and surgical consult. A basic metabolic panel should be ordered to evaluate and correct electrolyte imbalances that may contribute to ileus	
Postoperative recommendation #4	
Postoperative routine administration of sugarless chewing gum is recommended	Quality of evidence: Low Recommendation Grade: Weak
Postoperative implementation strategy #4	
Postoperative routine administration of sugarless chewing gum thrice daily starting on POD#0 is recommended	

POD = postoperative day.

The demonstrated benefits of ERAS in other surgical procedures and the lack of current recommendations regarding the anterior approach underscores the need to develop protocols which specifically address the complexities of standalone and ALIF 360s. The unique approach and anatomy involved in ALIF encompasses several surgical specialties, including general, vascular, and spinal surgery. It also carries its own set of complications that differentiate it significantly from posterior lumbar fusion surgeries. We took the first step towards closing this information gap by assembling a cross-cutting, multidisciplinary workgroup to develop protocol recommendations based on an evidence-based consensus process as well as implementation strategies for our health system. The goal was to define protocol recommendations that could be implemented across a variety of disciplines involved in the care of ALIF patients to achieve the best possible outcomes.

Our workgroup reviewed a large number of studies. Where spine and ALIF-specific research was lacking, articles from other surgical areas and expert opinions were reviewed, including those in general, colorectal, vascular, and general orthopedic surgery. Therefore, recommendations where the quality of evidence was moderate or high in these non-spine specialties with similar considerations were likewise graded as moderate or high evidence and strong recommendations. We felt this was justified because the ALIF procedure incorporates elements of general, colorectal, vascular, and spine surgery. We also included weak recommendations with low-quality evidence in a few scenarios. One, when expert opinion within the group based on anecdotal experience strongly supported its adoption and/or the risk of harm was considered to be negligible, such as is the case with our recommendations for postoperative lumbar brace, maintenance of desired intraoperative mean arterial pressures, and postoperative chewing gum to stimulate bowel function.

It is also important to keep in mind that our literature review was not exhaustive, therefore additional literature could exist which could potentially upgrade or downgrade the quality of evidence and the associated grades of our recommendations. Furthermore, many of the details included in the implementation strategies for each recommendation were not supported by evidence, and therefore relied heavily on the expert opinion of our workgroup. Therefore, we suggest providers looking to adapt and implement these recommendations modify them according to their organizational practices.

This initiative has revealed numerous research gaps and opportunities. Broadly, these gaps need to be filled to strengthen low-quality or no evidence protocol recommendations; quantitatively prove or disprove the associations between protocol recommendations and outcomes; and to provide additional evidence specific to the implementation strategies outlined herein.

For example, pain control is a critical part of ERAS protocols in other surgical specialties and it has been demonstrated that spinal surgery is associated with significant amounts of pain in the immediate postoperative period.^[20]^ Research is needed to determine whether the pre-, intra-, and postoperative multimodal analgesic approaches outlined within these recommendations are associated with decreased pain and a lower requirement for opiates, increased patient satisfaction, and other short- and long-term outcomes. In addition, the treatment for PONV in the setting of ALIF needs to be further studied. Research evaluating the relationships between improved surgical outcomes and prehabilitation, rehabilitation, early mobilization, and lumbar braces in spinal surgery is also lacking. Additionally, the protocol recommendations and implementation strategies should be graded using patient-reported outcomes. These patient-based metrics, which are usually performed in the outpatient setting, provide useful information to both the providers and their patients. They are critical for evaluating short- and long-term surgical outcomes, help guide the appropriateness and efficiency of a treatment strategy, and demonstrate the value of the strategy.^[287]^

## 4. Conclusions

Our next steps involve realizing the implementation strategies, and investigating how it improves surgical and patient-reported outcomes. This will be done both retrospectively and prospectively. Additionally, involvement of the ERAS^®^ Society in the refinement of these recommendations and potential development of official guidelines will be critical to encourage broader dissemination and implementation of ALIF best practices beyond our institution. We propose that the results of our multidisciplinary initiative to recommend and specify strategies for best practices in ALIF patients within our health system is a major step that will ultimately improve the overall outcomes for ALIF patients globally.

## Acknowledgements

The authors thank Anne Murray, PhD, MWC^®^ of the Clinical Research Institute at Methodist Health System for providing editorial support.

## Author contributions

**Conceptualization:** Richard Meyrat, Elaina Vivian, Archana Sridhar, R. Heath Gulden, Sue Bruce, Amber Martinez, Lisa Montgomery, Donald Reed, Peter J. Rappa, Hetendra Makanbhai, Kenneth Raney, Jennifer Belisle, Stacey Castellanos, Judy Cwikla, Kristin Elzey, Kristen Wilck, Fallon Nicolosi, Michael E. Sabat, Chris Shoup, Randall B. Graham, Stephen Katzen, Bartley Mitchell, Michael C. Oh, Nimesh Patel.

**Data curation:** Richard Meyrat, Elaina Vivian, Archana Sridhar, Sue Bruce, Kenneth Raney, Nimesh Patel.

**Formal analysis:** Richard Meyrat, Elaina Vivian, Archana Sridhar, R. Heath Gulden, Sue Bruce, Amber Martinez, Lisa Montgomery, Donald Reed, Peter J. Rappa, Hetendra Makanbhai, Jennifer Belisle, Stacey Castellanos, Judy Cwikla, Kristin Elzey, Kristen Wilck, Fallon Nicolosi, Michael E. Sabat, Randall B. Graham, Stephen Katzen, Bartley Mitchell, Michael C. Oh.

**Methodology:** Richard Meyrat, Elaina Vivian, Archana Sridhar, Sue Bruce.

**Project administration:** Richard Meyrat, Elaina Vivian, Archana Sridhar, Sue Bruce, Chris Shoup.

**Resources:** Chris Shoup.

**Supervision:** Richard Meyrat, Elaina Vivian, Sue Bruce.

**Writing – original draft:** Richard Meyrat, Elaina Vivian, Archana Sridhar, R. Heath Gulden, Sue Bruce, Amber Martinez, Lisa Montgomery, Donald Reed, Peter J. Rappa, Hetendra Makanbhai, Kenneth Raney, Jennifer Belisle, Stacey Castellanos, Judy Cwikla, Kristin Elzey, Kristen Wilck, Fallon Nicolosi, Michael E. Sabat, Chris Shoup, Randall B. Graham, Stephen Katzen, Bartley Mitchell, Michael C. Oh, Nimesh Patel.

**Writing – review & editing:** Richard Meyrat, Elaina Vivian, Archana Sridhar, R. Heath Gulden, Sue Bruce, Amber Martinez, Lisa Montgomery, Donald Reed, Peter J. Rappa, Hetendra Makanbhai, Kenneth Raney, Jennifer Belisle, Stacey Castellanos, Judy Cwikla, Kristin Elzey, Kristen Wilck, Fallon Nicolosi, Michael E. Sabat, Chris Shoup, Randall B. Graham, Stephen Katzen, Bartley Mitchell, Michael C. Oh, Nimesh Patel.

## Supplementary Material
