# Supplementary material for: Development of multidisciplinary, evidenced-based protocol recommendations and implementation strategies for anterior lumbar interbody fusion surgery following a literature review
Source: Medicine (Baltimore). 2023 Nov 24;102(47):e36142. doi: 10.1097/MD.0000000000036142 (PMC10681460; doi:10.1097/MD.0000000000036142)
Supplement: Supplementary file 1 [file medi-102-e36142-s001.docx]

Table. Thirty-Nine Appropriate Protocol Recommendations for Anterior Lumbar Interbody Fusion Surgery

| **Domain** | **No.** | **Recommendation** | **Appropriateness Ratings Round 1, Round 2** |
| --- | --- | --- | --- |
|  | **Patient education and counselling** | |  |
| Preoperative | 1 | Preoperative patient education is recommended. | 9, 9 |
| Postoperative | 2 | Discharge education is recommended. |  |
|  | **Prehabilitation** | |  |
| Preoperative | 3 | Prehabilitation combining exercise, nutritional therapy and psychological preparation is recommended. | 9, 9 |
|  | **Preoperative smoking cessation** | |  |
| Preoperative | 4 | Smoking cessation interventions are recommended at a minimum of four weeks before surgery. | 9, 9 |
|  | **Preoperative alcohol cessation** | |  |
| Preoperative | 5 | Alcohol cessation interventions are recommended at a minimum of four to eight weeks before surgery to reduce postoperative complications. | 9, 9 |
|  | **Preoperative diabetes monitoring** | |  |
| Preoperative | 6 | Diabetes monitoring with HbA1c and glucose levels in high- risk patients is recommended. | 9, 9 |
|  | **Preoperative computed tomography angiography of aorta and iliac vessels** | |  |
| Preoperative | 7 | Preoperative computed tomography angiography (CTA) of aorta and iliac vessels is recommended in patients with a history of stroke, peripheral arterial disease (PAD), or smoking. | 9, 9 |
|  | **Preoperative fasting and oral carbohydrate load** | |  |
| Preoperative | 8 | Preoperative reduced fasting is recommended. | 9, 9 |
| Preoperative | 9 | Preoperative administration of oral carbohydrate load before induction of anesthesia may be considered | 8, 9 |
|  |  | | |
| Preoperative | 10 | Preoperative risk assessment for postoperative nausea and vomiting (PONV) and administration of prophylactic antiemetic regimen based on risks is recommended. | 9, 9 |
| Intraoperative | 11 | A multimodal approach using TIVA while limiting volatile anesthetics and opioids where possible is recommended. Give corticosteroids and an antiemetic during surgery to prevent PONV. | 9, 9 |
| Postoperative | 12 | Postoperative rescue with a different class of antiemetic for patients with persistent PONV is recommended. | 9, 9 |
|  | **Antimicrobial prophylaxis** | |  |
| Preoperative | 13 | Preoperative decolonization of methicillin-sensitive and methicillin-resistant S. aureus is recommended for all patients. | 7.5, 9 |
| Intraoperative | 14 | Intraoperative broad-spectrum IV antibiotics covering *S. aureus* and skin preparation with an alcohol-based iodine or chlorhexidine solution is recommended | 7.5, 9 |
|  | **Patient Warming** | |  |
| Preoperative | 15 | Preoperative core temperature monitoring and pre-warming before induction of anesthesia is recommended for all patients undergoing ALIF. | 9, 9 |
| Intraoperative | 16 | Intraoperative patient core temperature should be monitored. Normothermia should be maintained through active warming of the patients. |  |
|  | **Fluid management** | | |
| Preoperative | 17 | Recommend liberalized fluid intake up to two hours before induction of anesthesia to achieve euvolemia. | 8, 9 |
| Intraoperative | 18 | Intraoperative IV fluid therapy using buffered isotonic crystalloids is recommended to maintain euvolemia in all patients undergoing ALIF. | 8, 9 |
| Postoperative | 19 | Postoperative IV fluids should be restricted once the patient starts oral intake, unless clinically indicated. | 8, 9 |
|  | **Management of urinary catheters** | | |
| Intraoperative | 20 | Urinary catheters when used should be removed postoperatively as soon as clinically indicated. | 9, 9 |
|  | **Surgical approaches** | | |
| Intraoperative | 21 | The retroperitoneal approach is recommended for ALIF surgeries. | 9, 9 |
|  | **Intraoperative neurophysiological monitoring** | | |
| Intraoperative | 22 | Intraoperative neurophysiological monitoring is recommended for all patients. | 9, 9 |
|  | **Intraoperative pulse oximetry of the left great toe** | | |
| Intraoperative | 23 | Intraoperative pulse oximetry of the left great toe is recommended during ALIF procedures | 9, 9 |
|  | **Intraoperative mean arterial pressure monitoring** | | |
| Intraoperative | 24 | Intraoperative mean arterial pressure should be maintained at a range of 70-100mm Hg is recommended. | 9, 9 |
|  | **Prophylaxis against venous thromboembolism** | | |
| Postoperative | 25 | Routine use of mechanical and chemoprophylaxis against venous thromboembolism is recommended for all patients. | 9, 9 |
|  | **Nutrition** | | |
| Preoperative | 26 | Preoperative nutritional screening and optimization of nutritional status is recommended for all patients undergoing ALIF. | 8.5, 9 |
| Postoperative | 27 | Recommend early oral clear liquid diet and advance as tolerated in the absence of postoperative nausea and vomiting for all patients. |  |
| Postoperative | 28 | Early postoperative high-protein diet is recommended. | 8.5, 9 |
|  | **Early mobilization** | | |
| Postoperative | 29 | Early mobilization is recommended for all patients | 9, 9 |
|  | **Rehabilitation** | | |
| Postoperative | 30 | Recommend acute care physical therapy and occupational therapy during hospitalization for all patients. Recommend outpatient physical therapy (PT) for all patients | 9, 9 |
|  | **Lumbar brace** | | |
| Postoperative | 31 | Consider using postoperative lumbar braces when mobilizing ALIF patients | 9, 9 |
|  | **Multimodal analgesia** | | |
| Preoperative | 32 | A preoperative multimodal opioid-sparing analgesia strategy is recommended | 9, 9 |
| Intraoperative | 33 | Minimize intra-operative use of opioids. Consider combining regional anesthetic blocks with general anesthesia to reduce intra- and postoperative opioid requirements. | 9, 9 |
| Postoperative | 34 | Multimodal opioid-sparing analgesic protocol for postop pain management should be used in all patients undergoing ALIF. | 9, 9 |
|  | **Postoperative ileus** | | |
| Preoperative | 35 | Preoperative routine administration of stool softeners is recommended. | 8.5, 9 |
| Postoperative | 36 | Routine use of postoperative stool softeners is recommended. | 9, 9 |
| Postoperative | 37 | Routine use of postoperative laxatives is recommended. | 9, 9 |
| Postoperative | 38 | Consider performing an ileus workup in patients with no signs of bowel movement, intolerance to oral diet, and/or obstipation despite treatment regimen for constipation. | 8, 9 |
| Postoperative | 39 | Postoperative routine administration of sugarless chewing gum is recommended. | 8, 9 |

Meyrat, R. Development of Multidisciplinary, Evidenced-Based Protocol Recommendations and Implementation Strategies for Anterior Lumbar Interbody Fusion Surgery following a Literature Review
